# Prediction of Sequential Organelles Localization under Imbalance using A Balanced Deep U-Net

**DOI:** 10.1038/s41598-020-59285-9

**Published:** 2020-02-14

**Authors:** Novanto Yudistira, Muthusubash Kavitha, Takeshi Itabashi, Atsuko H. Iwane, Takio Kurita

**Affiliations:** 1 Hiroshima University, Department of Information Engineering, Higashi Hiroshima, 739-8521 Japan; 2 Riken, Center for Biosystems Dynamics Research, Laboratory for Cell Field Structure, Higashi Hiroshima, 739-0046 Japan; 3 Hiroshima University, Graduate School of Integrated Sciences for Life, Higashi Hiroshima, 739-0046 Japan; 4 Osaka University, Graduate School of Frontier Biosciences, Osaka, 565-0871 Japan; 5 Universitas Brawijaya, Fakultas Ilmu Komputer, Malang, 65145 Indonesia

**Keywords:** Image processing, Machine learning

## Abstract

Assessing the structure and function of organelles in living organisms of the primitive unicellular red algae *Cyanidioschyzon merolae* on three-dimensional sequential images demands a reliable automated technique in the class imbalance among various cellular structures during mitosis. Existing classification networks with commonly used loss functions were focused on larger numbers of cellular structures that lead to the unreliability of the system. Hence, we proposed a balanced deep regularized weighted compound dice loss (RWCDL) network for better localization of cell organelles. Specifically, we introduced two new loss functions, namely compound dice (CD) and RWCD by implementing multi-class variant dice and weighting mechanism, respectively for maximizing weights of peroxisome and nucleus among five classes as the main contribution of this study. We extended the Unet-like convolution neural network (CNN) architecture for evaluating the ability of our proposed loss functions for improved segmentation. The feasibility of the proposed approach is confirmed with three different large scale mitotic cycle data set with different number of occurrences of cell organelles. In addition, we compared the training behavior of our designed architectures with the ground truth segmentation using various performance measures. The proposed balanced RWCDL network generated the highest area under the curve (AUC) value in elevating the small and obscure peroxisome and nucleus, which is 30% higher than the network with commonly used mean square error (MSE) and dice loss (DL) functions. The experimental results indicated that the proposed approach can efficiently identify the cellular structures, even when the contour between the cells is obscure and thus convinced that the balanced deep RWCDL approach is reliable and can be helpful for biologist to accurately identify the relationship between the cell behavior and structures of cell organelles during mitosis.

## Introduction

**Figure 1:**
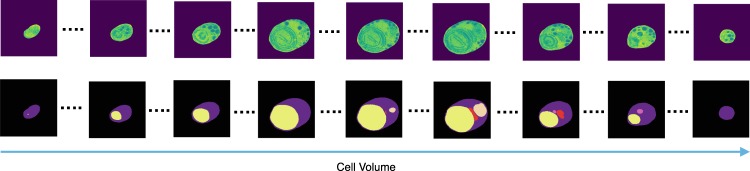
Sequential FIB-SEM images of organelles in *C*. *merolae* cell during cell division. First row presents stained sequential images of cell body immediately after cytokinesis. Second row shows its corresponding ground truth indicating cytoplasma (purple), plastid (yellow), mitochondrion (red), nucleus (light brown), and peroxisome (pink).

The interpretation of live cells captured over extended periods using an advanced scanning electron microscopy (SEM) is useful for identifying sequential cell behavior and local changes based on its environment^1,2^. However, different stages of organelles in the primitive unicellular red algae *Cyanidioschyzon merolae* (*C*. *merolae*) cell during cell division appear in various position and time^3^ and thus subjective evaluation of sequential cell contours undergoing cell division is tedious and challenging (Fig. 1). Advancement in microscopy and imaging with spatial and temporal resolution is highly helpful to determine the location of cell patterns to assess the biological cell information. Thus objective evaluation of accurate position of cell contours using translation invariant technique is beneficial to monitor the sequential cell cycle for mitosis. Moreover, several automatic methods have been developed to address the mitotic events, morphology and their count in various pathological studies^4–6^.

Mainly, automated approaches used handcrafted features^6–8^ and image processing techniques^9^ for binary cell image segmentation. However, the occurrences of large variations of texture patterns make the handcrafted technique difficult and not feasible for accurate segmentation of cells. Advancement of classification accuracy and availability of high computational power^10,11,^ the cell image segmentation gradually shifted to deep convolutional neural network (CNN). The deep CNN^12^ has shown its potential ability in image segmentation tasks with non-handcrafted features. Several CNN architectures for yeast cell classification system used cleaned and patch-wise cell data set to train their network^4,5,13^. However, different types of multi-class microscopy and staining images usually comprised of unequal distribution of cell classes and it can significantly minimize the performance of the classifier. Particularly Unet-based CNN^14^ architecture has been developed with pixel-wise weighting objective function to address the imbalance cell class problem in binary cell image segmentation. Moreover, to identify specific morphology under temporal data, the number of pixels in each class is not balanced over time which in turn leads to performance reduction and hence the weighting mechanism based on the overlapped cell boundary is not appropriate for monitoring the sequential cells with class imbalanced condition. It is a primary challenge when automating the multi-class cell image segmentation.

Moreover, automatic analysis of sequential cell segmentation is still lack of discussion and mostly treat it as a temporal problem rather than imbalanced data set problem. In various medical applications, Unet-based models with casual and generalized dice loss functions have been shown better performance than simple CNN model, still prone to misclassification in the imbalanced environment^15–21^. Here, with the focus of developing an automatic system for recognizing the multi-label problem in which each image slice consists of different cell regions that belong to different classes of organelles, this study automatically updating the weights of the imbalanced classes by constructing a new objective function. Because of the localization of five different cellular regions with obscure boundaries along with its non-cellular textures, it is needed to design a network with appropriate objective function which maximizes weights on the whole image regarding lower instance cell regions that help direct increase of number of true positives. Specifically, we proposed two new loss functions; compound dice loss (CDL) and regularized weighted compound dice loss (RWCDL) functions in the Unet-like CNN model to replace the previous loss functions for better segmentation of cell organelles. The main contribution of this study can be summarized as follows: (i) proposed a balanced deep RWCDL function for localizing sequential cell organelles on FIB-SEM images for mitosis; (ii) investigated the potential of our proposed loss functions with commonly used loss function for improved detection; (iii) designed architectures in recognizing five classes (cytoplasma, plastid, nucleus, mitochondrion, and peroxisome) are compared to ground truth segmentation in terms of various performance metrics.

## Materials

### Image data acquisition

The primitive unicellular red algae, *C*. *merolae*, which is considered to be the oldest eukaryotic organisms with mitochondria has simple individual double-membrane organelles such as the nucleus, mitochondrion and plastid and single-membrane organelles such as peroxisome, ER, Golgi complex, etc. The size of the cell is 2–5 micrometer and the sequential 2D electron microscopy images (average of 300–500) were obtained for structural modeling of organelles at whole cell level. A single cell perfectly divides into two daughter cells like mammalian cells. *C*. *merolae* cells (NIES-1804) were obtained from National Institute for Environmental Studies and cultured in 2x Allen’s medium under continuous light (50 W/m2 square meter) at 42 degrees. Cells were harvested at the log phase. After physical fixation of the cells, staining the *C*. *merolae* was resin-embedded. It is stained by using osmium and heavy metals. The sequential SEM images were obtained using focused ion beam-SEM (FIB-SEM), which provides 3D structural information of a whole cell^2^. Sequential SEM image stack (serial block face:SBF) is processed and aligned using “Amira” ver5.6 software (Thermo Fisher Scientific inc.). A single-cell volume data from SBF is possible to be picked up from the image by cropping as visualized in Fig. 1. All images gathered from SEM were carefully analyzed and labeled by experts. The cells are labeled based on its structural segments of cytoplasma, plastid, nucleus, mitochondrion, and peroxisome^22^.

## Method

### Data enhancement

**Figure 2:**
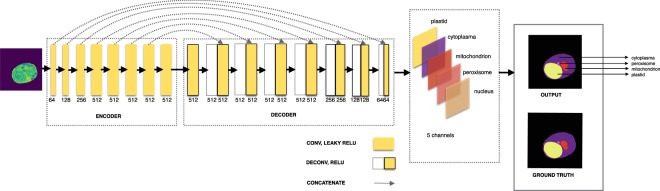
Proposed deep balanced network architecture using regularized weighted compound dice loss for localizing cell organelles during mitosis.

Here we proposed an automatic approach using Unet-like deep CNN architecture to localize the structure of five different classes of organelles for monitoring the cells (Fig. 2). However, directly training an original cell image along with its non-cell regions in deep network is not appropriate for accurate localization of cell regions. Hence, we enhanced the training set with simple pre-processing steps. The cell image is cropped to remove the non-cell regions. The cropped image is then converted into grayscale image followed by resizing into 256 × 256 pixels using bilinear interpolation.

### Architecture design for cell organelles localization

The incomplete image features of patch-based training are not appropriate for the multi-label imbalanced data set segmentation. To reduce the variations in learning cell structures we feed whole image as input into the segmentation network. The proposed deep Unet-like CNN architecture (Fig. 2) consists of encoder and decoder path. It has one channel of input and five channels of output. For encoder path, we used CNN that includes eight convolutional layers, each layer followed by leaky ReLU. As shown in Table 1, the encoder paths reduce the feature map by 2. Each convolutional layer has 3 × 3 or 4 × 4 kernels with batch normalization operation starting from second convolution. The number of channels starts from 64 and doubles until it reaches 512. The number of channels in the following layers does not change. The output channels for the eight encoder path include 64, 128, 256, 512, 512, 512, 512, and 512, respectively. For decoder path, we used seven upsampling deconvolution layers, each of which is followed by ReLU. The deconvolutions, all of which use batch normalization, decrease the number of channels by half except the first one. The output of an upsampling deconvolution is concatenated with an output of the corresponding part of the encoder. The output channels for the eight upsampling deconvolutions of decoder include 512, 512, 512, 512, 512, 256, 128, and 64, respectively. The last part of decoder is 2D convolution. The final output of 5 channels indicates the five classes of organelles. The total number of parameters reaches nearly 54 M.

**Table 1:** Proposed architecture design with parameters (K, O, I, W, H are kernel size, output channel, input channel, output width, and output height, respectively).

Network Path	Layer	Size of *K* × *K* × *O* × *I*	Output Size (*O* × *W* × *H*)	Batch Normalization	Activation	Dropout	#Parameters
Encoder	conv1	3 × 3 × 64 × 1	64 × 256 × 256	False	Leaky ReLU	False	640
	conv2	4 × 4 × 128 × 64	128 × 128 × 128	True	Leaky ReLU	False	131,200
	conv3	4 × 4 × 256 × 128	256 × 64 × 64	True	Leaky ReLU	False	524,544
	conv4	4 × 4 × 512 × 256	512 × 32 × 32	True	Leaky ReLU	False	2,097,152
	conv5	4 × 4 × 512 × 512	512 × 16 × 16	True	Leaky ReLU	False	4,194,816
	conv6	4 × 4 × 512 × 512	512 × 8 × 8	True	Leaky ReLU	False	4,194,816
	conv7	4 × 4 × 512 × 512	512 × 4 × 4	True	Leaky ReLU	False	4,194,816
	conv8	4 × 4 × 512 × 512	512 × 2 × 2	True	Leaky ReLU	False	4,194,816
Decoder	deconv1	4 × 4 × 512 × 512	512 × 4 × 4	True	ReLU	True	4,194,816
	deconv2	4 × 4 × 512 × 1024	512 × 8 × 8	True	ReLU	True	8,389,120
	deconv3	4 × 4 × 512 × 1024	512 × 16 × 16	True	ReLU	True	8,389,120
	deconv4	4 × 4 × 512 × 1024	512 × 32 × 32	True	ReLU	False	8,389,120
	deconv5	4 × 4 × 256 × 1024	256 × 64 × 64	True	ReLU	False	4,194,432
	deconv6	4 × 4 × 128 × 512	128 × 128 × 128	True	ReLU	False	1,048,704
	deconv7	4 × 4 × 64 × 256	64 × 256 × 256	True	ReLU	False	262,208
	conv	3 × 3 × 5 × 128	5 × 256 × 256	False	—	False	5,765
Total							54,406,469

The mean square error (MSE) loss is calculated for cell region segmentation by using Eq. (1)

1 $$MSE(k)=\frac{1}{K}\,\mathop{\sum }\limits_{k=1}^{K}\,\mathop{\sum }\limits_{n=1}^{N}\,{({s}_{nk}-{\hat{p}}_{nk})}^{2}$$

where ${\hat{p}}_{nk}$ is the predicted probability, which assigns a probability or activation value to each class *k* for each pixel *n* and ${s}_{{n}_{k}}$ is ground truth label. The index *k* iterates over the number of organs and *n* over the number of input image pixels. The parameters *K* represents the number of classes and *N* is number of pixels of an image. Therefore, the output is an average of multiple binary MSE values. However, it remains difficult to reduce false-positive predictions for small objects with imbalanced number of classes. Therefore, to improve the learning on not well segmented classes, we proposed two new loss functions; compound dice (CD) and regularized weighted compound dice (RWCD).

#### Compound dice loss

The classical dice similarity coefficient (DSC) is an overlap index that is widely used to asses the segmentation mapping^23^. The two-class dice loss (DL)^24^ is used to maximize the overlapping area between the predicted and the ground truth. It is expressed as

2 $${L}_{Dice}=1-\frac{2\,{\sum }_{i=1}^{I}\,{\sum }_{j=1}^{J}\,{s}_{ij}{p}_{ij}+m}{{\sum }_{i=1}^{I}\,{\sum }_{j=1}^{J}\,{s}_{ij}+{\sum }_{i=1}^{I}\,{\sum }_{j=1}^{J}\,{p}_{ij}+m}$$

where *s*_*ij*_ and *p*_*ij*_ represent ground truth and prediction at each pixel with *i* row and *j* column, respectively. To prevent division by zero, the smoothing parameter *m* is added to both the denominator and numerator. In DL, weights are allocated to different segmentation labels based on the number of training data and thus it equally weighs the locations of false positives (FP) and false negatives (FN). It creates unstable optimization in the severely imbalanced data set. As a result the high false positive results is still focused on the true positive region. Hence in order to reduce the number of false positives, we introduced compound dice loss (CDL) as a weighted sum of two-class variant dice loss, which can be expressed as

3 $${L}_{CD}=1-\frac{2\,{\sum }_{i=1}^{I}\,{\sum }_{j=1}^{J}\,{s}_{ij}{p}_{ij}+m}{{\sum }_{i=1}^{J}\,{\sum }_{j=1}^{J}\,{s}_{ij}+{\sum }_{i=1}^{I}\,{\sum }_{j=1}^{J}\,{p}_{ij}+m}+\frac{2\,{\sum }_{i=1}^{I}\,{\sum }_{j=1}^{J}\,{({s}_{ij}-{p}_{ij})}^{2}+m}{{\sum }_{i=1}^{I}\,{\sum }_{j=1}^{J}\,{s}_{ij}+{\sum }_{i=1}^{I}\,{\sum }_{j=1}^{J}\,{p}_{ij}+m}$$

The smoothing parameter (*m*) we used in Eq. 2&3 are 1.0 by experiment. The proposed CDL function not only forces the inner joint intersection but also minimizes the outer joint which maximizes the number of true positives while minimizing the number of false positives. Hence, it is straight forward in creating flexibility and balancing of both FPs and FNs. We calculated multi-class CDL as the accumulation of all classes using the following equation

4 $${L}_{CD}=\mathop{\sum }\limits_{k}^{K}\,{L}_{C{D}_{k}}$$

where *k* indicates output class channel for each class. The parameter *K* represents the total number of classes.

#### Regularized weighted loss

The variations of class distribution makes the model heavily focus on the over represented classes. Alternatively, additional weighting mechanism is needed to increase the instances of under represented classes. The traditional weighted loss function is based on mean square error weighting on each class based on the presence of target class pixels^25^. However, in multi-label joint object segmentation, the data sets do not consist of all occurrences of classes poorly influences the activation in the following layers and sometimes it leads to zero gradient updates in the back-propagation process. Therefore, if either foreground or background pixels are zero, then the weights *w* become zero. In order to improve the detection rate of TPs in multi-label joint object segmentation, we proposed a very precise multi-class regularized weighted loss (RWL) function which calculates the image-wise pixel weights. It is defined as the summation of each class weights by approximating the number of foreground pixels from the total number of pixels of target class, which is inversely proportional to the labels frequency. It takes the form

5 $${w}_{k}=1-\frac{{N}_{fk}}{{N}_{fk}+{N}_{bk}}$$

where *w*_*k*_ indicates weight per class *k*. The parameters *N*_*fk*_ and *N*_*bk*_ represent the number of foreground and background pixels of each class *k*, respectively. In addition, we evaluated the combined regularized weighted compound dice loss (RWCDL) function for the segmentation of cell structures of organelles. It computes the summation of class weight *w*_*k*_ multiplied with ${L}_{C{D}_{k}}$, where *k* indicates output channel of each class. It is expressed as:

6 $${L}_{RWCD}=\mathop{\sum }\limits_{k}^{K}\,{w}_{k}{L}_{C{D}_{k}}$$

The network designed with *L*_*RWCD*_ function can make the model efficient by maximizing appropriate weights of true positives regarding lower instance cell regions and, at the same time, it can control the model parameters from getting stuck in local minima by the multi-class variant dice loss. Hence, it can efficiently handle the FPs and FNs over the whole image.

## Experimental Settings

### Experiment

We evaluated three grouping of sequential cell volume data sets. Each grouping cells consists of five stages of mitotic process, in which each stage includes five classes of cytoplasma (C), plastid (Pl), nucleus (N), mitochondrion (M), and peroxisome (Pe). The stage indicated a part of whole mitotic cycle. Hereafter we refer three grouping as G-1, G-2 and G-3 data sets, which included 5047, 3078 and 1507 for training, and 5047, 3080 and 1500 for testing, respectively. The number of stages of G-1, G-2 and G-3 are varies from 5 (stage I to V) to 3 (stage I, II, IV). We used total number pixels of each class in training dataset. The number of occurrences of C, Pl, N, M, and Pe in G-1 (2931, 1950, 1147, 938 and 543, respectively), G-2 (1616, 1022, 745, 528, and 337, respectively) and G-3 (798, 460, 273, 205 and 109, respectively) are different among each other in the data sets. The data set with the proportion of 50:50 is selected randomly for training and testing the model. For training, the model iterates through 200 epochs for G-1 and 100 epochs for G-2 and G-3 data set. The number of iterations varies depending on the size of the training data set. Adam^26^ is used as an optimizer with hyperparameter of exponential decay rate of first order moment, exponential decay rate of second order moment, epsilon, schedule multiplier, and weight decay in decoder of 0.5, 0.999, *e*^−8^, 1.0, and 0.00001, respectively. We set the initial learning rate of 0.0001 for CDL and RWCDL. The initial learning rate depends on the convergence of each loss function and the learning rate is progressively reduced by multiplying 0.9 for every 1000 iterations. A sigmoid activation with threshold of 0.8 was used to output the network using DL, CDL and RWCDL functions. A threshold with 0.5 was used to output the network using MSE loss. All networks were implemented in python using Chainer framework with 12 GB memory of NVIDIA GTX TITAN.

### Evaluation

We evaluated original and proposed loss functions in the Unet-like deep CNN architecture for the segmentation of five classes of organelles on sequential SEM cell images. The efficiency of the architectures in predicting the five classes of cell contour is compared to the ground truth segmentation. We compared three architectural prediction performances in localizing C, Pl, N, M, and Pe based on MSE, DL, our proposed CDL and RWCDL functions. We compared the performance of both qualitative and quantitative results of all architectures. The quantitative networks performance was measured using the average value of precision, recall, F1 score, and area under the curve (AUC)^27^. The image which does not contain the true labels of any five classes is not included in the evaluation because the zero value of true positives (TP) influences the average value of AUC and F1-score. AUC metric is used to measure the performance of probability map under various thresholds. If the predicted class region belongs to the valid ground truth region, then it is considered a TP, whereas in other class occurrences, it is considered as a false positive (FP). If the predicted class region is correctly identified but does not belong to the valid ground truth region; then, it is considered a true negative (TN). We carried out additional experiments to show the ability and reliability of the proposed deep balanced RWCDL network to address the multi-label imbalance problem on G-3 dataset. To indicate the superiority of the proposed network in identifying lower number of instances of classes, we compared the performance of the RWCDL network with different existing dice loss functions such as generalized dice coefficient (GDC)^23^, dice plus binary cross entropy (D + BCE)^24^ and focal dice (FD)^28^ networks under various number of ratios of class distributions. We used *γ* of 2.0 and *α* of 0.2 to compute FD. To show the reliability and generalization ability of the proposed balanced RWCDL network in elevating under represented class, we investigated five-fold cross-validation method, which randomly partitioning the datasets into k parts (k = 5) with different members of the training and the testing data. The mean and standard deviations of these five different compositions of all five classes prediction performance of our proposed CDL and RWCDL networks were compared with the MSE and DL based networks performance.

## Results

### Comparison of networks performance on G-1 dataset

**Figure 3:**
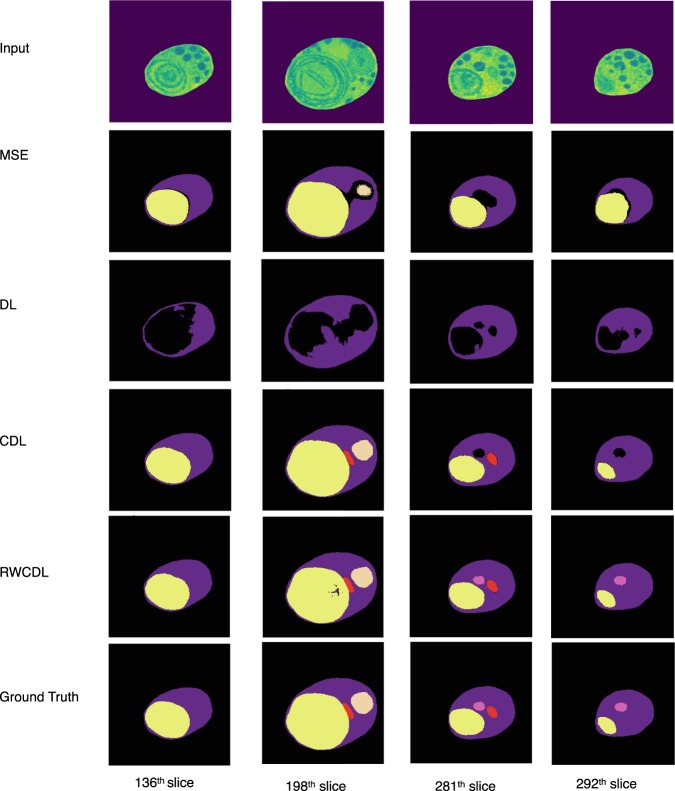
Visual comparison of our proposed network using regularized weighted compound dice loss with proposed compound dice loss, generalized dice loss and mean square error loss in localizing organelles at successive slices.

The visualization of few representative examples of segmentation of organelles at different slices based on MSE, DL, CDL and RWCDL are presented in Fig. 3. Among all the three groups of data sets evaluated in this study, the number of occurrences of peroxisome class is very less compared to the remaining four classes. The peroxisome class is indicated as pink color in the visualization results and it is considered as the highly imbalanced class in this study (Fig. 3). The comparison of networks performance statistics on G-1 data set is presented in Table 2. The proposed network with original MSE shows highest performance with high precision (90.0%) in identifying cytoplasma among other classes. As the number of occurrences decreases, the network using MSE function showed lesser potential. Moreover in Table 2 the architecture with MSE produces zero F1 score in recognizing the nucleus, mitochondrion and peroxisome classes. Similarly the network with DL function also produces zero F1 score in recognizing all classes except cytoplasma. It is because the less number of instances of cell structures are insufficient to provide additional capacity to train the network. Furthermore, the binary class of DL function is not sufficient for multi-class cell segmentation given the number of five segmented cells in this study. However, accurately detecting the minority instances of classes is often important for monitoring the cell structures. The segmentation performance of under represented classes is improved in the network with our proposed CDL function, especially in identifying nucleus, mitochondrion and peroxisome. Though the overall precision of our proposed CDL is almost similar with MSE network, the recall, F1 score and AUC is much higher than the MSE network. The improvement is also shown in the qualitative results with the appearance of mitochondrion, which is failed to detect with the MSE based network. Compared to the network with CDL function, the network with RWCDL function showed highest performance in detecting the imbalanced peroxisome class. The ranges of precision, recall, F1 score and AUC using RWCDL is almost 4% to 7% higher than using CDL. It is also true in the visual results of appearing the peroxisome cell contour using RWCDL function in the network.

**Table 2:** Comparison of performance metrics of deep Unet-like CNN architectures on G-1 data set.

Loss function	Class	Precision	Recall	F1 Score	AUC
*L*_*MSE*_	Cytoplasma	**0.9070**	0.8912	0.8867	0.9412
	Plastid	0.8896	0.8794	0.8727	0.9369
	Nucleus	0.0000	0.0000	0.0000	0.5000
	Mitochondrion	0.0000	0.0000	0.0000	0.5000
	Peroxisome	0.0000	0.0000	0.0000	0.5000
	overall	**0.8963**	0.7742	0.8192	0.8856
*L*_*Dice*_	Cytoplasma	0.8938	0.9011	0.8877	0.9447
	Plastid	0.0000	0.0000	0.0000	0.5000
	Nucleus	0.0000	0.0000	0.0000	0.5000
	Mitochondrion	0.0000	0.0000	0.0000	0.5000
	Peroxisome	0.0000	0.0000	0.0000	0.5000
	overall	0.8938	0.5139	0.6289	0.7558
*L*_*CD*_	Cytoplasma	0.9053	0.9231	0.9074	0.9563
	Plastid	0.8898	**0.9018**	**0.8813**	**0.9475**
	Nucleus	0.7922	0.6708	0.6966	0.8344
	Mitochondrion	0.8097	**0.7410**	**0.7437**	**0.8687**
	Peroxisome	0.7284	0.5798	0.6100	0.7896
	overall	0.8940	**0.8836**	**0.8842**	**0.9400**
*L*_*RWCD*_	Cytoplasma	0.9029	**0.9300**	**0.9087**	**0.9599**
	Plastid	**0.8924**	0.8992	0.8750	0.9460
	Nucleus	**0.8156**	**0.6848**	**0.7158**	**0.8414**
	Mitochondrion	**0.8287**	0.7125	0.7406	0.8549
	Peroxisome	**0.7963**	**0.6468**	**0.6827**	**0.8232**
	overall	0.8935	0.8823	0.8827	**0.9400**

### Comparison of networks performance on G-2 dataset

The comparison of networks performance statistics on G-2 data set demonstrated that the network learning with our proposed CDL function obtained higher recall (79.6%), F1 score (80.3%), and AUC (89.3%) than the network learning with original MSE and DL functions (Table 3). Whereas, the CDL function is not enough to produce a better performance in the imbalanced class of peroxisome, it showed low F1 score value of 18.2%. Still it needs to push the trade off between the TP and FP. The network with the proposed combined RWCDL function outperformed on G-2 data set with the AUC value of 80.4% in predicting peroxisome, which is 23% higher than the CDL and 30% higher than the MSE function networks.

**Table 3:** Comparison of performance metrics of the proposed Unet-like CNN architectures on G-2 data set.

Loss function	Class	Precision	Recall	F1 Score	AUC
*L*_*MSE*_	Cytoplasma	0.8272	0.8942	0.8343	0.9352
	Plastid	0.8018	0.7261	0.7343	0.8585
	Nucleus	0.5944	0.4206	0.4678	0.7090
	Mitochondrion	0.0000	0.0000	0.0000	0.5000
	Peroxisome	0.0000	0.0000	0.0000	0.5000
	overall	0.8333	0.7480	0.7846	0.8706
*L*_*Dice*_	Cytoplasma	0.8267	0.9341	0.8615	0.9531
	Plastid	0.0000	0.0000	0.0000	0.5000
	Nucleus	0.0000	0.0000	0.0000	0.5000
	Mitochondrion	0.0000	0.0000	0.0000	0.5000
	Peroxisome	0.0000	0.0000	0.0000	0.5000
	overall	0.8268	0.5336	0.6270	0.7642
*L*_*CD*_	Cytoplasma	0.7990	0.9189	0.8328	0.9432
	Plastid	0.8200	0.7073	0.7305	0.8493
	Nucleus	0.7356	0.6794	0.6821	0.8360
	Mitochondrion	0.6948	0.4729	0.5216	0.7341
	Peroxisome	0.3233	0.1545	0.1821	0.5763
	overall	0.8120	0.7963	0.8030	0.8934
*R*_*WCD*_	Cytoplasma	**0.8467**	**0.9425**	**0.8784**	**0.9590**
	Plastid	**0.8933**	**0.8058**	**0.8286**	**0.8989**
	Nucleus	**0.8391**	**0.7114**	**0.7326**	**0.8540**
	Mitochondrion	**0.7789**	**0.6436**	**0.6772**	**0.8194**
	Peroxisome	**0.7381**	**0.6081**	**0.6355**	**0.8037**
	overall	**0.8701**	**0.8625**	**0.8657**	**0.9863**

### Comparison of networks performance on G-3 dataset

The comparison of networks performance statistics on G-3 data set also demonstrated that the network learning based on RWCDL function is comparatively higher in classifying all five classes with high AUC values compared over CDL, DL and MSE functions (Table 4). The highest AUC value for the segmentation of peroxisome in G-1, G-2 and G-3 data sets using MSE, DL, CDL and RWCDL networks is 50.0%, 50.0%, 78.9% and 82.3%, respectively. Hence, trained with our proposed loss functions, the network is able to achieve significant balance in the imbalanced classes. These encouraging results suggested that the proposed deep balanced RWCDL learning function based on pixel–wise weights could be beneficial to improve the multi-class joint cell structure segmentation results. The experimental results of the five-fold cross-validation of the performance of RWCDL and CDL networks are compared with MSE and DL networks in segmenting five classes of organelles is presented in Table 5. The overall mean performance results of all classes using five-fold cross validation for our proposed CDL and RWCDL were much higher than those of the MSE and DL. In particular, the performance of our proposed CDL and RWCDL networks in evaluating the values of precision, recall, F1 score and AUC produced almost similar ranges in detecting the organelle classes, the balanced RWCDL network achieved higher performance in locating the minority peroxisome class. The precision, recall, F1 score and AUC of RWCDL is 0.6106, 0.3330, 0.3633, and 0.6661, respectively in locating the minority peroxisome class, which is much higher than those values 0.4628, 0.2985, 0.3325, and 0.6490 for CDL, indicating the proposed balanced RWCDL network is reliable and can able to achieve significant balanced schema in such a highly imbalanced cell structure distributions.

**Table 4:** Comparison of performance metrics of the proposed Unet-like CNN architectures on G-3 data set.

Loss function	Class	Precision	Recall	F1 Score	AUC
*L*_*MSE*_	Cytoplasma	**0.8728**	0.8949	0.8673	0.9410
	Plastid	0.8386	0.7468	0.7571	0.8702
	Nucleus	0.7830	0.6208	0.6606	0.8090
	Mitochondrion	0.3602	0.2167	0.2611	0.6079
	Peroxisome	0.0000	0.0000	0.0000	0.5000
	overall	0.8624	0.7951	0.8220	0.8953
*L*_*Dice*_	Cytoplasma	0.7944	0.8986	0.8158	0.9355
	Plastid	0.0000	0.0000	0.0000	0.5000
	Nucleus	0.0000	0.0000	0.0000	0.5000
	Mitochondrion	0.0000	0.0000	0.0000	0.5000
	Peroxisome	0.0000	0.0000	0.0000	0.5000
	overall	0.7949	0.5333	0.6227	0.7641
*L*_*CD*_	Cytoplasma	0.8607	0.9499	0.8910	0.9676
	Plastid	0.8528	0.7777	0.7748	0.8855
	Nucleus	0.8583	0.7206	0.7595	0.8595
	Mitochondrion	**0.8191**	0.6592	0.7080	0.8291
	Peroxisome	**0.7350**	0.4463	**0.5313**	**0.7320**
	overall	0.8876	0.8604	0.8730	0.9906
*L*_*RWCD*_	Cytoplasma	0.8611	**0.9567**	**0.8962**	**0.9706**
	Plastid	**0.8749**	0.7775	**0.7881**	**0.8862**
	Nucleus	**0.8649**	0.7432	**0.7771**	**0.8704**
	Mitochondrion	0.8038	**0.6872**	**0.7177**	**0.8431**
	Peroxisome	0.7185	**0.4644**	0.5087	0.7229
	overall	**0.8927**	**0.8668**	**0.8788**	**0.9906**

**Table 5:** Five-fold cross-validation of the performance of our proposed network using regularized weighted compound dice loss with proposed compound dice loss, generalized dice loss and mean square error loss in localizing organelles on G-3 dataset.

Loss function	Precison	Recall	F1 Score	AUC
*L*_*MSE*_	0.8605 ± 0.0163	0.7156 ± 0.0207	0.7698 ± 0.0131	0.8559 ± 0.0101
*L*_*Dice*_	0.7606 ± 0.0964	0.5352 ± 0.0413	0.6102 ± 0.0164	0.7637 ± 0.0178
*L*_*CD*_	0.8653 ± 0.0162	0.8499 ± 0.0141	0.8566 ± 0.0143	0.9223 ± 0.0073
*L*_*RWCD*_	0.8648 ± 0.0119	0.8461 ± 0.0118	0.8544 ± 0.0117	0.9204 ± 0.0061

Furthermore, the comparison of networks under different class ratios were demonstrated that the network learning based on RWCDL function is comparatively higher in classifying all five classes with background pixels (third to seventh row) indicated highest F1 scores compared over GDC, D + BCE, and FD (Table 6). Specifically, the balanced RWCDL network using a large number of training instances (eighth row) and highly imbalanced ratios with additional background only images outperforms other networks in terms of precision, recall and F1 score values, represented the reliability of the proposed network in elevating the imbalanced class distributions. Figure 4 demonstrated the stability of the proposed RWCDL network under different ratios of foreground and background pixels. The class ratio was calculated based on the number of foreground pixels divided by number of background pixels. Intuitively, the farther the ratio from 1 the more imbalance foreground/background occurs in the dataset. We use mean accuracy as parameter to measure the performance. Mean accuracy considers balanced performance of foreground and background segmentation, of which is defined as (*TPR* + *TNR*)/2 where *TPR* and *TNR* are true positive rate and true negative rate, respectively. It shows that the RWCDL is significantly stable and consecutively increasing the performance especially in the highly imbalanced decreased ratios of class distribution.

**Table 6:** Performance comparisons of our proposed network using regularized weighted compound dice loss over generalized dice coefficient, dice plus binary cross entropy and focal dice networks under various number of ratios of class distributions in localizing organelles on G-3 dataset.

#Training Instances	Class Ratios						GDC			D + BCE			FD			Proposed RWCDL		
	C	Pl	N	M	Pe	BG	Precision	Recall	F1score	Precision	Recall	F1score	Precision	Recall	F1score	Precision	Recall	F1score
597	0.27	0.18	0.17	0.12	0.27	—	**0.6639**	0.4855	0.5433	0.6492	0.5420	0.5890	0.6088	**0.5988**	0.5851	0.6547	0.5663	**0.5991**
1230	0.29	0.20	0.16	0.21	0.13	—	0.6618	**0.5673**	0.6063	0.6068	0.5001	0.5455	0.6108	0.5521	0.5736	**0.6820**	0.5635	**0.6365**
1614	0.31	0.21	0.21	0.16	0.1	—	0.6204	**0.7951**	0.6900	0.6898	0.6124	0.6460	0.7342	0.6665	0.6955	**0.7840**	0.6847	**0.7290**
2212	0.36	0.29	0.16	0.12	0.07	—	0.7348	**0.7735**	0.7570	0.8317	0.6898	0.7496	0.7837	0.7177	0.7460	**0.8841**	0.7578	**0.8118**
2410	0.41	0.26	0.15	0.11	0.07	—	0.7016	0.7493	0.7233	0.7814	0.6649	0.7143	0.8005	0.6723	0.7223	**0.8585**	**0.8379**	**0.8465**
3107	0.26	0.21	0.11	0.08	0.05	0.22	0.6313	0.7189	0.6684	0.8288	0.7440	0.7810	0.8066	0.6237	0.6817	**0.9160**	**0.8230**	**0.8648**

**Figure 4:**
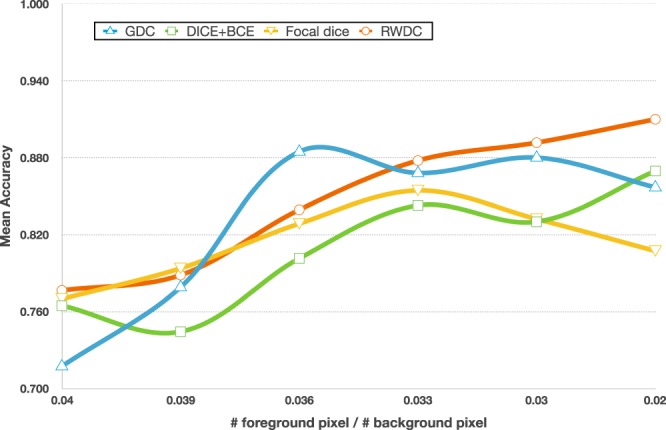
Comparison of mean accuracy values of networks using various number of foreground-background ratios of class distributions based on our proposed regularized weighted compound dice loss, generalized dice coefficient (GDC), dice plus binary cross entropy (D + BCE) and focal dice (FD) on G-3 dataset. Note that the training data size increases as the foreground to background pixel ratio increases.

## Discussion

This study proposed a balanced, deep regularized weighted compound dice loss (RWCDL) function for investigating the five classes of cell organelles on FIB-SEM images. Our proposed RWCDL and CDL functions training behavior in recognizing the cell organelles is demonstrated in Unet-like CNN architectures. In particular, the localization of small ROI with fewer instances of classes across the large ROI class regions is a key challenge in this study. The network trained with commonly used MSE-based and DL-based objective functions lead erroneous results in the segmentation of multi class cellular regions that appeared in various position along with its non cell textures. However, our proposed loss functions revealed relatively higher performance, even with a high level of imbalances for localizing the peroxisome. Moreover, the network trained with combined RWCDL function outperformed the network trained with CDL function. The advantage of this combined loss function is that it can able to push the model weights to the appropriate balance for TP pixels and, at the same time, maintain the model parameters do not get stuck in local minima.

The CNN-based learning structures with usual cross entropy objective functions have been used to classify on microscopy images often generated biased predictions and misleading accuracy in identifying multi-class cellular regions^4,5,13^. A simple CNN model for multi-class classification of yeast cell images achieved the highest average precision value of 84.0%^5^. A multi instance learning CNN model on yeast cell protein images delivered an average precision of 85.5%^4^. Similarly, the overall results of our proposed deep balanced approach also demonstrated superior performance with highest precision of 89.3%. The per cell localization of high throughput microscopy yeast cell image with CNN network stated high accuracy of 91.0%^13^, whereas the highest overall AUC of our balanced deep network is 99.1%. However, aforementioned CNN approaches with usual objective function found difficulties and delivered poor detection rate in localizing peroxisome region because the patch-wise segmentation network can lead to loss of information when handling with lower instances cell patterns. Therefore, the model accuracy may concentrate only on majority instance cell patterns and thus creating the non-reliability of the system. However, to achieve full coverage to the targeted regions, the proposed network used whole image-wise weighting technique. Hence, differing from above approaches, we investigated encoder-decoder CNN architecture with our newly proposed balanced RWDCL that is able to identify the lower instance peroxisome in almost all cells, even when the contour between the cells is obscure. The balanced approach can efficiently increase the weight of the peroxisome and gained a robust localization rate with highest AUC value of 82.3%, which is almost similar in classes with large number of instances experimented in this study.

The choice of loss function is a crucial component for better segmenting the dense cell regions. It is concurred in the recent work of mitosis event detection, who found the network with combined embedding loss functions performed better than the commonly used loss functions^29^. Our experimental results of balanced network trained using combined RWCDL function outperformed the other designed networks, especially in localizing regions with lower number of instances. Additionally, the commonly used Unet with *L*_*MSE*_ and *L*_*Dice*_ models with sequential SEM cell image delivered poor performance with the range of 50.0–60.0% and 60.0%, respectively, of the AUC score in detecting mitochondrion and peroxisome regions. Overall, the promising results of our proposed balanced approach showed the wide applicability of distinguishing between the contours of the obscure cells and thus confirms the system reliability. Though our approach of multi-cell localization proves its capability of annotating almost all cells and performed better than the other designed architectures, it relatively focused on weighting pixels based on the foreground and background of each class. There is space for further improvement of the proposed loss functions and parameter tuning. For further work, it would be interesting to adapt long short-term memory in CNN for better understanding of sequence information of mitosis process. In addition we will extend the proposed balanced automated approach on 3D structural models of organelles at the whole cell level for robust segmentation of large scale *C*. *merolae* cell during cell division.

## Conclusions

With the goal of developing an automated system, we proposed a deep balanced network with RWCDL function to localize the multi-class cell organelles on FIB-SEM image. We investigated our proposed loss functions training behavior in Unet-like CNN architectures. Compared to our proposed loss functions using compound dice, the combined regularized weighted compound dice efficiently addressed the lower instances classes such as mitochondrion, nucleus, and peroxisome localization, by pushing desirable weight between false positives and false negatives while minimizing to local minima at the same time. Based on the experimental results and validations we have shown the reliability and efficiency of our proposed balanced approach especially in distinguishing obscure and low instance cellular patterns that helps biologist to make a reliable decision throughout the mitotic process for understanding the function and behavior of the sequential cells.
